# Toward Personalized Medicine: The Utility of Enthesis and Joint Ultrasound Examination in Defining the Phenotype of Patients with Acute Anterior Uveitis

**DOI:** 10.3390/jpm16060297

**Published:** 2026-05-31

**Authors:** Giorgia Citriniti, Filippo Crescentini, Niccolò Possemato, Nicolo Girolimetto, Alessandra Rai, Elena Bolletta, Pietro Gentile, Luca De Simone, Fabrizio Gozzi, Chantal Adani, Maria Grazia Orlando, Luca Cimino, Carlo Salvarani, Pierluigi Macchioni

**Affiliations:** 1 Rheumatology Department, AUSL-IRCCS Azienda Unità Sanitaria Locale-Istituto di Ricovero e Cura a Carattere Scientifico di Reggio Emilia, 42123 Reggio Emilia, Italy; filippo.crescentini@ausl.re.it (F.C.); niccolo.possemato@ausl.re.it (N.P.); alessandra.rai@ausl.re.it (A.R.); mariagrazia.orlando@ausl.re.it (M.G.O.); carlo.salvarani@ausl.re.it (C.S.); pierluigi.macchioni@ausl.re.it (P.M.); 2 Internal Medicine & Rheumatology, Azienda Unità Sanitaria Locale, Bologna-Istituto di Ricovero e Cura a Carattere Scientifico, Azienda Universitaria Ospedaliera Bologna, 40138 Bologna, Italy; nicolo.girolimetto@ausl.re.it; 3 Ocular Immunology Unit, Azienda Unità Sanitaria Locale-Istituto di Ricovero e Cura a Carattere Scientifico, 42123 Reggio Emilia, Italy; elena.bolletta@ausl.re.it (E.B.); pietro.gentile@ausl.re.it (P.G.); luca.desimone@ausl.re.it (L.D.S.); fabrisio.gozzi@ausl.re.it (F.G.); chantal.adani@ausl.re.it (C.A.); luca.cimino@ausl.re.it (L.C.); 4 Department of Surgery, Medicine, Dentistry and Morphological Sciences, University of Modena and Reggio Emilia, 41124 Modena, Italy; 5 Rheumatology Unit, University of Modena & Reggio Emilia, 41121 Modena, Italy

**Keywords:** acute anterior uveitis, ultrasound entheseal examination, spondyloarthritis

## Abstract

**Background/Objectives**: Patients with acute anterior uveitis (AAU) have a high prevalence of occult spondyloarthropathy (SpA). Only one previous study investigated ultrasonographic (US) changes at peripheral entheses and joints in patients with acute anterior uveitis (AAU) and no study has used these data in recognizing unknown SpA as defined by ASAS criteria. No previous study compared non-granulomatous (NGAU) with granulomatous uveitis (GAU) for these US features. The objective of this study was to investigate the prevalence of US enthesis and joint abnormalities in a consecutive series of GAU and NGAU patients and to use these data in recognizing unknown SpA as defined by ASAS criteria. **Methods**: A total of 152 consecutive patients diagnosed with AAU [103 NGAU (42 B27−, 61 B27+) and 49 GAU] from the Immunology Eye Unit (AUSL-IRCCS Reggio Emilia, Italy) were enrolled in this study. Six peripheral entheses as well as knee and ankle joints were bilaterally evaluated by US according to standard methods. The presence of any elementary lesion, structural damage and active enthesitis, according to OMERACT definitions, was recorded. ASAS classification criteria of SpA were integrated with the US data of active enthesitis and joint synovitis in defining the presence of enthesitis and arthritis. **Results**: NGAU patients had a higher prevalence of entheseal erosions (11.9% vs. 9%, P0 0.009), of enthesis exhibiting a PD signal (29.7% vs. 12.2%, *p* = 0.025) and of active enthesitis (23% vs. 8.2%, *p* = 0.026) compared with GAU group. Unknown SpA as defined by ASAS criteria was recognized in 29 patients by clinical and MR/Rx examination of sacroiliac joints and in 13 additional patients with the use of US data. **Conclusions**: Acute entheseal lesions and erosions are associated with NGAU, without apparent influence of HLA-B27 positivity. US examination helps to recognize previously unknown SpA and to define the disease phenotype of patients, moving toward more personalized treatment.

## 1. Introduction

Spondyloarthritis (SpA) is a family of inflammatory rheumatic diseases that includes ankylosing spondylitis (AS), psoriatic arthritis (PA), arthritis associated with inflammatory bowel disease (IBD), reactive arthritis (formerly known as Reiter’s syndrome), and undifferentiated SpA [1].

These conditions are characterized by various combinations of articular synovitis, spondylitis, dactylitis and enthesitis, frequently associated with extra-musculoskeletal manifestations [1].

Among these manifestations, acute anterior uveitis (AAU) is the most common, with a reported prevalence ranging between 15% and 33% according to meta-analysis [2,3,4]. AAU is also the most frequent type of uveitis worldwide, particularly in Western countries, where it accounts for approximately 50–92% of all uveitis cases [5]. Recent epidemiological studies have reported that AAU represents approximately 49–58% of uveitis cases in Italy [6,7]. From a clinical perspective, distinguishing between non-granulomatous anterior uveitis (NGAU) and granulomatous anterior uveitis (GAU) is mandatory for correct diagnostic evaluation [8]. Most cases of NGAU are associated with autoimmune diseases, primarily spondyloarthritis (SpA) [9], whereas GAU, such as Fuchs’ uveitis, is generally not associated with SpA.

Several studies have evaluated the prevalence of previously undiagnosed SpA among consecutive series of patients presenting with AAU. Prevalence varies widely depending on imaging methods, diagnostic criteria, and patient selection. Based on clinical evaluation alone, the prevalence of SpA among AAU patients has been reported to range from 20% (newly diagnosed cases) [10,11] to as high as 55% and 76% in HLA-B27+ patients [12,13]. When magnetic resonance imaging (MRI) of the sacroiliac joints (SIJs) is included in the diagnostic work-up, the prevalence of undiagnosed SpA is 22% and 56% in unselected AAU patients and in those reporting chronic back pain, respectively. The prevalence of SpA also depends on the diagnostic criteria applied (ASAS classification criteria or expert opinion) [14,15,16,17]. In one study, radiographic evaluation of the sacroiliac joints in patients with chronic low back pain lasting more than three months and with onset before the age of 45 years revealed a prevalence of axial spondyloarthritis of 63% when both definite and probable diagnoses were considered [18].

In a recent retrospective study conducted in our Ocular Immunology Unit, we estimated the prevalence of concomitant SpA in a series of consecutive patients with NGAU to be 36%.

AAU may be the first manifestation of upcoming Spa, and early identification of patients at risk is crucial for early diagnosis and appropriate treatment.

AAU generally precedes the onset of arthritis in a substantial proportion of patients (45.1% and 33.3% in AS and PsA, respectively) [19]. In addition, in a multivariable model, longer diagnostic delay (>5 years) has been associated with a higher incidence of uveitis (hazard ratio [HR] 4.01; 95% CI:3.23, 4.07) and IBD events (HR 1.85; 95% CI: 1.28, 2.67) in patients with AS [19].

Enthesitis is considered the hallmark of SpA and may represent the earliest manifestation of the disease [20]. However, clinical assessment of enthesitis may be challenging [21], while musculoskeletal ultrasound has consistently been shown to be more sensitive than clinical examination in detecting entheseal involvement.

In 2018, using a consensus-based stepwise approach, a standardized US definition and scoring system for enthesitis in SpA was developed for use in clinical studies [22]. SpA-related enthesitis definition includes several elementary lesions such as hypoechogenicity, thickening of the tendon insertion, calcifications, enthesophytes, erosions and, if active, a Doppler signal within 2 mm from the bony cortex.

Among these components, the Doppler signal appears to be the most specific and most representative ultrasound feature of active entheseal inflammation in SpA [23].

Several studies have evaluated the diagnostic value of PDUS at entheseal sites in populations at high risk of developing SpA, including patients with recent inflammatory back pain (IBP) suggestive of axSpA [24,25,26].

Other studies have identified subclinical enthesitis in patients with SpA-related diseases such as psoriasis [27,28,29], IBD [30,31,32] and axial SpA [24,33,34,35]. These studies observed a low concordance rate between clinical and US findings with a high prevalence of entheseal abnormalities at US examination, even in the absence of clinically overt enthesitis or arthritis. To date, only one previous study has evaluated the presence of subclinical enthesitis and/or arthritis detected by US in AAU [36] and the potential role of US in identifying previously undiagnosed peripheral SpA.

The primary objective of this study was to evaluate the prevalence of clinical signs and ultrasonographic findings of enthesitis and arthritis in a consecutive series of patients with NGAU and GAU seen in ophthalmology outpatient clinics, stratified according to uveitis type and HLA-B27 positivity. In addition, we evaluated the prevalence of previously undiagnosed SpA in this cohort according to ASAS classification criteria [37,38] using clinical, radiological (MR and or Rx of the sacroiliac joints) and US-detected inflammatory entheseal and joint features as surrogate for clinical involvement.

## 2. Materials and Methods

This was a prospective monocentric study conducted in collaboration with the Ocular Immunology Unit and the Rheumatology Clinic of the AUSL-IRCCS of Reggio Emilia, Italy, between January 2016 and January 2019. The study was approved by the local ethics committee according to current Italian legislation on epidemiological studies (2016/0100242).

The study population consisted of adult patients (age 18–65 years) diagnosed by the ophthalmologist with non-infectious acute anterior uveitis (AAU) consecutively seen by the Ocular Immunology Unit. The anatomical classification and final diagnosis were based on the criteria of the International Uveitis Study Group (IUSG) and the Standardization of Uveitis Nomenclature (SUN) Working Group [39,40]. AAU cases were categorized as granulomatous anterior uveitis (GAU, Fuchs’ uveitis) or non-granulomatous anterior uveitis (NGAU) according to clinical features. Written informed consent was obtained from all participants in accordance with the Declaration of Helsinki. Exclusion criteria for further evaluation included the presence of other identifiable causes of uveitis.

All patients underwent a complete ophthalmic evaluation, including best-corrected visual acuity (BCVA), intraocular pressure (IOP), slit-lamp examination of the anterior segment with clinical assessment of anterior chamber inflammation, and the fundus after pupillary dilatation.

Blood tests including serum C-reactive protein CRP, erythrocyte sedimentation rate (ESR) and HLA-B27 typing were performed in all patients. Collected data included demographic characteristics, anthropometric measures (including body mass index), lifestyle factors (including alcohol consumption, smoking habits and physical activity), social profile, date of disease onset and diagnosis, concomitant diseases, and ongoing and previous medications. After identification by the ophtalmologist, patients were evaluated by a rheumatologist expert in SpA (NP, NG, FC).

The rheumatological assessment included a structured interview covering family history of arthritis, SpA, psoriasis, IBD, uveitis and other inflammatory rheumatic conditions. Past medical history of previous arthritis (pain and swelling in one or more joints), peripheral enthesitis (defined as severe pain and limited function lasting at least 2 weeks at an anatomical enthesis, particularly in the calcaneal region), and dactylitis (a history of a sausage digit) and the presence of chronic back pain, inflammatory back pain (according to ASAS criteria [41]), buttock pain and its distribution, and anterior chest wall pain were also evaluated. Clinical rheumatological examination included joint count (66 joints were evaluated for swelling and 68 for tenderness) as well as enthesitis count; the presence and location of dactylitis sites and measures of finger-floor distance and Schober’s test were also recorded. The following entheses were examined for tenderness and swelling bilaterally: common extensor tendon (CET) insertion on the lateral epicondyle of the humerus, quadriceps tendon (QT), patellar tendon (PT), tibial tuberosity (TT), Achilles tendon (AT) and plantar fascia (PF) insertion on the calcaneus. The Leeds Enthesitis Index (LEI) [42] and Maastricht Ankylosing Spondylitis Enthesitis Score (MASES) [43] were calculated. The Bath Ankylosing Spondylitis Disease Activity Index (BASDAI) [44] and Bath Ankylosing Spondylitis Functional Index (BASFI) [45] were also recorded. Patients were excluded if they had tendinitis related to overuse, mechanical stress or recent joint injury. Additional exclusion criteria included intra-articular or peri-entheseal corticosteroid injections in the previous four weeks, enrollment in other clinical trials and any medical condition that could have limited their ability to participate in the study.

Ultrasound examination was performed in B-mode and PD-mode using an ESAOTE MyLabClassC, Genoa, Italy) equipped with an 18-6 MHz and 13-5 MHz multifrequency linear probe operated by two rheumatologists (PM, GC) experienced in musculoskeletal ultrasonography and blinded to diagnosis and clinical findings. All images were recorded digitally. US evaluation was performed on the same day of the ophthalmological and rheumatological assessment. The following entheseal sites were examined bilaterally in transverse and longitudinal planes according to the EULAR-OMERACT recommendation [46]: CET, QT, PT, TT, AT, and PF. In B-mode, the following abnormalities were recorded according to OMERACT definitions [22,47]: entheseal thickening, entheseal hypoechogenicity, bony erosions, enthesophytes/calcification, and enlargement of bursae. Each lesion was scored dichotomously as present or absent (1 or 0).

Entheses were scored globally according to the following: the presence or absence of lesions, acute involvement (according to the OMERACT definition of the presence of hypoechoic increased thickness of anatomical enthesis (at enthesis tendon insertion < 2 mm from the bony surface) exhibiting a Doppler signal) [22], active involvement according to DiMatteo [23], defined as (1) power Doppler (PD) at the enthesis grade ≥ 1 plus entheseal thickening and/or hypoechoic areas, or (2) PD grade > 1 (independent of the presence of entheseal thickening and/or hypoechoic areas), and chronic involvement (presence of >0 chronic lesion). PD signal was assessed using a pulse repetition frequency of 750 Hz and a PD gain of 50 to 53 dB. PD signal at the enthesis level was scored according to OMERACT definitions (Balint et al., 2019) [22]. The Madrid sonography enthesitis index (MASEI) [48] and Glasgow ultrasound enthesitis scoring system (GUESS) [33] were employed.

Knees and ankles were evaluated for the presence of synovial hypertrophy, joint effusion, and bone erosions. The presence of a PD signal and synovial hypertrophy were recorded using a 4-point semiquantitative scale (0 = absent, 1 = mild, 2 = moderate, 3 = severe). Flexor and extensor tendons of the foot were also evaluated at the ankle and dorsal foot regions for tendon sheath synovial hypertrophy, fluid distension, and Doppler signal according to EULAR recommendations [49].

The ultrasound examinations were performed jointly by the two rheumatologists, and the sonographic scores were determined by consensus.

The images collected from the examinations of patients with an active PD signal were reviewed to calculate intra-reader reproducibility.

Patients with inflammatory back pain underwent MRI of the sacroiliac joints to assess the presence of sacroiliitis according to ASAS classification criteria. MRI scans were evaluated by a radiologist with experience in musculoskeletal diseases (LS).

Spondyloarthritis was classified according to the ASAS classification criteria as axial or peripheral SpA [50,51]. Patients were divided into three groups: NGAU with newly diagnosed SpA (SpA+ new), NGAU with a previous diagnosis of SpA (SpA+ previous), and NGAU without SpA (SpA−). Patients were also stratified according to HLA-B27 positivity (B27+ vs. B27−). The presence of active entheseal lesions was considered indicative of enthesitis (clinical and subclinical), and patients were classified accordingly as fulfilling ASAS criteria.

### Statistical Analysis

Continuous variables were expressed as the mean and standard deviation (mean ± SD) or as the median and interquartile range (IQR), as appropriate. Categorical variables were reported as absolute frequencies and percentages. Continuous variables were compared using Student’s *t*-test or the Mann–Whitney U test when distributions were non-normal. Categorical variables were compared using the chi-square test or Fisher’s exact test, as appropriate. Intra-rater reliability was calculated using the intraclass correlation coefficient (ICC) for single measures. An ICC value > 0.50 was considered good and above 0.80 excellent. A *p*-value < 0.05 was considered statistically significant. Statistical analyses were performed using SPSS version 29.0 (IBM Statistics, Armonk, NY, USA).

## 3. Results

### 3.1. Demography

The final study population included 152 patients: 103 with NGAU and 49 with GAU. Demographic, clinical and laboratory findings and comorbidities of the study population are reported in Table 1.

Among patients with NGAU, 61 (60%) patients were HLA-B27-positive, whereas none of the patients with GAU carried the HLA-B27 gene. Statistically significant differences were found between NGAU and GAU groups for disease duration (*p* = 0.004) and serum CRP levels (*p* = 0.019).

Previous systemic steroid and DMARD treatment szx more frequently reported in NGAU patients (30.4% versus 8.2%, *p* = 0.002; and 12.7% versus 2%, *p* = 0.037, respectively), whereas the prevalence of any current therapy was not statistically different in the two groups.

### 3.2. Clinical Rheumatological Evaluation

As reported in Table 2, patients with NGAU had a significantly higher prevalence of a positive history of IBP, arthralgia, peripheral arthritis, and at least one musculoskeletal manifestation, as compared with patients with GAU.

A family history of ankylosing spondylitis and SpA was more frequently reported in patients with NGAU compared with those with GAU. Dactylitis was reported only in the NGAU group (7%) and almost exclusively among NGAU-B27+ patients, although the differences did not reach statistical significance because of the small number of cases.

Finally, a higher prevalence of rheumatological manifestation and family history of ankylosing spondylitis was observed when comparing B27+ and B27- patients within the NGAU group.

At least one painful joint at examination was more frequently observed in NGAU patients compared to GAU patients. No patients presented dactylitis at objective examination.

Clinical evidence of enthesitis (tenderness and/or swelling) was observed in 37.5% of the total AAU patients, with no significant differences between NGAU and GAU patients. The mean number of painful entheses was statistically significantly higher in NGAU vs. GAU patients. 

MASES values and the proportion of patients with a score of at least one were higher in the NGAU group than in the GAU group. No differences were observed for LEI in NGAU-B27+ and NGAU-B27− patients. No statistically significant differences in arthritis/enthesitis/dactylitis prevalence were observed when comparing patients with shorter vs. longer disease duration (<12 months versus >12 months).

Overall, 44% of patients with NGAU fulfilled ASAS classification criteria for axial and/or peripheral SpA (40% and 28%, respectively). Statistically significant differences were observed in the prevalence of patients fulfilling ASAS criteria when comparing NGAU and GAU patients (*p* < 0.001), with no patient in the GAU group fulfilling ASAS criteria. Similar significant differences were observed when comparing B27+ and B27− NGAU patients. No differences were observed in the proportion of patients fulfilling ASAS criteria according to disease duration (<12 months versus >12 months), although a higher proportion of ASAS-positive patients were observed in the long disease duration group (41% versus 33%, *p* = 0.433).

### 3.3. Ultrasound Examination

At least one ultrasound (US) entheseal abnormality (acute or chronic) was detected in all patients (Table 3).

Significant differences between NGAU and GAU patients were observed for the mean number of entheses with erosions per patient, the prevalence of patients with at least one enthesis with erosions, the mean number of power Doppler (PD)-positive entheses, the proportion of patients with at least one PD-positive enthesis, and the mean number of active entheseal lesions per patient.

When comparing HLA-B27-positive and HLA-B27-negative patients within the NGAU group, only the mean number of entheses with structural damage differed significantly (Table 3).

At PD-mode evaluation, at least one abnormality was detected in 24.2% of the total AAU population, with a statistically significant difference between NGAU and GAU patients (*p* = 0.025). The mean number of entheses per patient showing a PD vascular signal was significantly higher in NGAU compared with GAU patients (*p* < 0.001).

Acute ultrasound signs of enthesitis were observed in 17 of the 152 patients, with a higher prevalence in the NGAU group; however, the difference between the two groups did not reach statistical significance (*p* = 0.058). Active enthesitis was significantly more frequent in the NGAU group than in the GAU group (*p* = 0.026). Similarly, the mean number of active entheses per patient was significantly higher in NGAU patients (*p* = 0.006) (Table 3).

Patients with NGAU had a significantly higher prevalence of active enthesitis at the tibial tuberosity (TT) compared with GAU patients (12.6% vs. 2%, *p* = 0.037). A higher, although not statistically significant, prevalence of active involvement was also observed at quadriceps tendon (QT) and lateral epicondyle (LE) entheses (8.7% vs. 4% and 11% vs. 2%, respectively). No differences were observed for active involvement at the remaining entheseal sites.

Chronic entheseal lesions were observed in 91.4% of patients overall, without significant differences between NGAU and GAU groups (*p* = 0.111) (Table 3). However, patients with at least one eroded enthesis were observed only in the NGAU group (*p* = 0.009), and the mean number of eroded entheses per patient was also significantly higher in this group (*p* < 0.001).

Mean values of GUESS and MASEI scores were significantly higher in NGAU than in GAU patients. No differences for the same scores were observed comparing NGAU-B27+ vs. NGAU-B27− patients (Table 3).

Forty-eight patients (31.6%) had knee abnormalities at US examination: 45 (29.6%) had joint effusion, 9 (6%) had synovial hypertrophy, and 6 (3.9%) had a synovial PD signal without a statistically significant difference in prevalence between GAU, NGAU, B27+ and B27− patient groups. No US alteration was observed at ankle joint examination.

The prevalence of ultrasound-detected tendon sheath abnormalities at the ankle and foot was negligible in the entire AAU population (<1%) without any difference between groups.

Table 4 reports the results of intra-rater ultrasound reliability for some selected variables comparing two subsequent evaluations of all recorded images of the 37 patients with a positive Doppler signal at entheseal sites. All ICC values are in the range of good or excellent concordance.

### 3.4. ASAS SpA Classification Criteria Implementation with Ultrasound Results

A total of 19 out of 103 (18%) NGAU patients had a previous diagnosis of SpA (axial 16 (84%), peripheral 3 (16%)) and were excluded from the subsequent analysis. No patient in the GAU group met ASAS classification criteria for SpA. After rheumatological examination (comprehensive of sacroiliac MRI and/or X-ray), 29 (35%) new patients fulfilled ASAS classification criteria for SpA. Considering US inflammatory features as surrogate of clinical entheseal and joint involvement, 13 (15%) more patients fulfilled ASAS SpA criteria, increasing the prevalence of unknown SpA from 35% to 50%. Twelve (86%) were HLA-B27-positive, two (14%) had associated IBD, twelve (86%) had US-detected active enthesitis and three (21%) had active synovitis at US knee examination.

## 4. Discussion

In this study, we demonstrated that unselected GAU and NGAU patients differ significantly in the prevalence of SpA-related clinical manifestations and family history of SpA-related diseases. As expected, previously diagnosed or newly identified SpA was significantly more frequent in the NGAU group, whereas no cases were observed in the GAU group. These differences were more pronounced when GAU patients were compared with HLA-B27-positive NGAU patients than with HLA-B27-negative patients. On ultrasound examination, signs of active or acute enthesitis, the presence of a power Doppler signal at the enthesis, and entheseal erosions were significantly more prevalent in NGAU patients than in GAU patients, whereas no differences were observed for other chronic or acute ultrasound characteristics. Similarly, NGAU HLA-B27-positive and NGAU HLA-B27-negative patients showed a comparable prevalence of ultrasound abnormalities, further suggesting that factors other than HLA-B27 may play a relevant role. To our knowledge, this is the first study evaluating rheumatologic manifestations and ultrasound abnormalities in a series of NGAU and GAU patients. The very low prevalence of these features in GAU patients underscores the importance of distinguishing GAU from NGAU in studies involving AAU patients, a distinction that has rarely been emphasized in previous research.

Several studies have evaluated the presence of rheumatologic clinical manifestations and ultrasound abnormalities in patients with SpA-related conditions, particularly psoriasis (PSO) [27,28,29] and inflammatory bowel disease (IBD) [30,31,32], with or without a previous diagnosis of SpA. These studies consistently demonstrated a significantly higher prevalence of clinical or ultrasound abnormalities compared with healthy controls. Muñoz-Fernández et al. [36] evaluated differences in ultrasound entheseal changes using the MASEI score [48] in different groups of AAU patients: their main finding was the absence of differences in MASEI scores between AAU patients with SpA and HLA-B27-positive AAU patients without SpA. They suggested that enthesopathy diagnosed at US examination in HLA-B27+ AAU patients may be considered part of an incomplete clinical form of SpA and that adding these data enables more patients to fulfill the Amor et al. criteria [51] for the classification of SpA.

Early diagnosis of SpA and appropriate treatment can significantly reduce disease morbidity and long-term functional disability [52,53,54]. Nevertheless, early recognition of SpA remains a major challenge because of the clinical heterogeneity of the disease and the high prevalence of extra-musculoskeletal manifestations [19]. Several studies have shown that AAU contributes to diagnostic delay in SpA and that occult SpA is frequently identified in AAU populations [19]. Most previous studies have relied on clinical evaluation, with or without imaging techniques such as MRI or radiography of the sacroiliac joints, to estimate the prevalence of previously undiagnosed SpA in AAU cohorts [10,11,12,13,14,15,16,17,18]. To date, however, no studies have systematically used ultrasound examination of entheses and joints to assess the prevalence of occult SpA in this patient population.

Ultrasound changes at the enthesis, particularly the presence of a power Doppler signal, have been proposed as potential preclinical indicators of impending SpA, as they are more frequently observed in patients with conditions predisposing to SpA or in the earliest stages of the disease. In this context, several referral strategies have been proposed to facilitate earlier recognition of SpA in patients presenting with AAU [10,14,17,55,56,57].

Algorithms proposed by Wach [10], Sykes [14] and Poddubnyy [57] include chronic back pain (duration ≥ 3 months) with onset before the age of 45 years as an entry criterion, whereas Van der Berg [55] considers chronic back pain regardless of age at onset. The DUET algorithm, proposed by Haroon et al, [56] includes chronic back pain or joint pain as entry criteria. These algorithms combine clinical features with HLA-B27 testing or imaging of the sacroiliac joints. Our findings are consistent with those of Rademacher et al. [17], who recommended referral to a rheumatologist for AAU patients presenting with musculoskeletal symptoms, given the atypical presentation of back pain in this population. Rai et al. have found that the combined use of MRI of the sacroiliac joints and ultrasound examination of entheses is useful to identify previously undiagnosed SpA in patients with NGAU.

At present, the predictive value of ultrasound-detected subclinical enthesitis for the development of SpA at the individual patient level remains uncertain.

Recent studies in psoriasis populations have shown that subclinical ultrasound alterations can be detected even in patients without clinical joint symptoms. For example, a meta-analysis demonstrated a higher prevalence of subclinical ultrasound abnormalities in psoriasis patients compared with healthy controls [58]. In a cohort of 547 psoriasis patients, 20.8% fulfilled CASPAR criteria for psoriatic arthritis, and 16.4% without clinical joint symptoms were classified as having subclinical PsA based on ultrasound findings [59].

Quadriceps tendon thickness [60], elevated baseline subclinical enthesitis, PDUS and GSUS synovitis score [61], structural enthesic lesions [62] or the presence of baseline active enthesitis [63] were found to be significant predictors of PsA development.

Several studies have reported that predominant structural abnormalities of the enthesis can be observed in healthy, elderly or obese subjects, or in patients with metabolic syndromes [64,65,66]. We have only utilized signs of inflammation as surrogate criteria for clinical involvement, thus reducing the possibility of misclassification.

Our study has several limitations, primarily related to the relatively small sample size. In addition, patients with a previous diagnosis of SpA were not excluded from the study, and some were receiving systemic treatments for their rheumatic disease. Moreover, some patients were treated with systemic corticosteroids or biologic agents for uveitis, which may have also reduced the detection of inflammatory findings at both clinical and ultrasound examination. Furthermore, only patients with self-reported chronic back pain underwent MRI or radiographic evaluation of the sacroiliac joints, leading to underestimation of the prevalence of undiagnosed axial SpA.

The clinical significance of US changes found in asymptomatic patients and their value in predicting progression to clinical SpA are currently unknown. The cross-sectional design of our study precludes assessment of the prognostic value of our findings, and longitudinal follow-up would be valuable in determining whether ultrasound-positive patients ultimately develop clinically overt SpA.

A major strength of this study is its single-center design, which ensured a homogeneous rheumatologic and ophthalmologic assessment. In addition, GAU patients were included as a control group alongside NGAU patients, a population that has not previously been investigated in this context. Furthermore, the inclusion of unselected patients irrespective of rheumatologic or genetic characteristics reflects real-life clinical practice and emphasizes the need for close collaboration between rheumatologists and ophthalmologists in the evaluation of AAU patients, as recently outlined by a recent publication of our group [67].

The causes of spondyloarthritis development in patients with acute anterior uveitis are largely unknown, but recognizing subclinical forms using ultrasound may allow for the early identification of patients at high risk of progression to overt clinical disease. Early diagnosis is essential for initiating appropriate therapy, and integrating clinical and imaging data allows for the development of targeted therapeutic strategies.

## 5. Conclusions

In a consecutive series of patients with GAU and NGAU, we demonstrated a high prevalence of clinical rheumatic conditions and entheseal and joint abnormalities on ultrasound exclusively in the NGAU group, and that ultrasound evaluation of entheses and joints can further improve their detection. In patients with AAU and suspected SpA, ultrasound evaluation of entheses and joints could therefore become part of the routine diagnostic work-up.

## Figures and Tables

**Table 1:** Demographic, clinical and laboratory findings of the patients at baseline evaluation.

	Total (*n* = 152)	GAU (*n* = 49)	NGAU (*n* = 103)	*p*	NGAU HLA-B27+ (*n* = 61)	NGAU HLA-B27− (*n* = 42)	*p*	GAU vs. NGAUB27+	GAU vs. NGAU B27−
Age (Y)	46 + 12	47.2 + 11.4	45.8 + 12.8	0.519	44.7 + 13	47.5 + 12	0.274	0.909	0.295
BMI	24.9 (5.1)	25.1 + 5.7	24.9 + 4.9	0.871	24.4 + 4.9	25.7 + 4.7	0.191	0.594	0.616
Female, *n* (%)	91 (61)	30 (61)	63 (61)	0.994	39 (64)	24 (57)	0.487	0.770	0.693
HLA-B27	61 (40)	0	61 (59)	<0.001	-	-	-	-	-
Age at diagnosis	41 + 13	38.4 (13.5)	42.5 (13.2)	0.063	40.3 (13.6)	45.6 (12.9)	0.043	0.007	0.413
Disease duration (m)	61.7 + 110.2	109 + 152	40 + 76	0.004	51.0 + 92.1	21.7 + 38.7	0.012	<0.001	0.025
<12 m	82	18 (36)	64 (63)	0.019	34 (56)	30 (71)	0.049	0.003	0.107
Acute		---	30 (29)	---	17	13	0.485	---	---
Recurrent uveitis		---	73 (71)	----	46	27		---	---
Bilateral		0	40 (40)	<0.001	22	18	0.357		
Hypopyon		0	2 (2)	0.327	2	0	0.253		
Synechiae		0	29 (28)	<0.001	12	17	0.011		
Cataract		29 (59)	19 (19)	<0.001	10	9	0.431		
Comorbidities									
Diabetes	3 (2%)	0	3 (3)	0.551	2 (3)	1 (2)	1.0	0.501	0.462
Dyslipidemia	36 (24%)	11 (22)	25 (24)	0.805	13 (21)	12 (29)	0.485	0.886	0.503
Hypertension	19 (12.5%)	5 (10)	14 (14)	0.612	6 (9)	8 (19)	0.180	1.0	0.248
Cardiovascular Diseases	3 (2%)	0	3 (2.9)	0.551	1 (2)	2 (3)	1.0	0.501	0.462
Psoriasis	11 (7.3)	1 (2)	10 (9.7)	0.106	4 (9.5)	6 (10)	1.0	0.129	0.177
Inflammatory bowel diseases	7 (4.7)	2 (4)	5 (5)	0.367	2 (2)	3 (5)	0.933	0.332	0.505
ESR (1st hour)	15.6 (14)	9.7 (6.9)	17.1 (14.9)	0.078	16.6 (14.1)	17.7 (16.2)	0.811	0.092	0.093
CRP (mg/dL)	0.51 (1.42)	0.18 (0.29)	0.65 (1.68)	0.019	0.79 + 1.91	0.45 + 1.22	0.380	0.042	0.223
CRP > 0.05 mg/dL	18.3%	2 (6.1)	18 (23.1)	0.034	5 (15.2)	13 (28.9)	0.183	0.018	0.427
Hb (g)	13.9 (1.15)				14.3 (1.1)	13.4 (1.2)	0.030	0.631	0.191
Previous topical steroids	142 (94)	43 (88)	99 (97)	0.058	60 (100)	39 (93)	0.067	0.007	0.498
Previous systemic steroids	35 (23)	4 (8)	31 (30)	0.002	21 (35)	10 (24)	0.277	<0.001	0.046
Previous dmards	14 (9)	1 (2)	13 (13)	0.037	12 (20)	1 (2)	0.013	0.006	1
Previous biological agents	7 (4.6)	0	7 (7)	0.097	5 (8)	2 (5)	0.697	0.063	0.210
Actual topical steroid usage	90 (60)	29 (59)	61 (60)	0.942	40 (67)	21 (50)	0.091	0.420	0.380
Actual systemic steroid usage	14 (9)	1 (2)	12 (12)	0.133	9 (15)	3 (7)	0.352	0.063	0.332
Actual dmard usage	4 (2.6)	0	4 (4)	0.305	4 (7)	0	0.141	---	0.126
Actual biological agent usage	6 (4)	0	6 (6)	0.178	6 (10)	0	0.041	---	0.032

Data are expressed as the mean (SD) or number (%). GAU: granulomatous acute uveitis; NGAU: non-granulomatous acute uveitis; BMI: body mass index; ESR: erythrocyte sedimentation rate; CRP: C-reactive protein; Hb: hemoglobin; DMARDs: disease-modifying antirheumatic drugs.

**Table 2:** Clinical rheumatological evaluation of patients.

	GAU (49 Patients)	NGAU (103 Patients)	*p*	NGAU HLA-B27+ (61 Patients)	NGAU HLA-B27− (42 Patients)	*p*	GAU vs. NGAUB27+	GAU vs. NGAUB27−
History								
Inflammatory Back Pain	5 (10)	40 (39)	<0.001 5.6; 2.0–15.3	28 (46)	12 (29)	0.078	<0.001 7.5; 2.6–21.4	0.032 3.5; 1.1–11.0
Arthralgia	3 (6)	28 (27)	0.002 5.7; 1.6–19.9	20 (33)	8 (19)	0.124	<0.001 7.5; 2.1–27.0	0.154
Peripheral arthritis	1 (2)	17 (16)	0.008 9.5; 1.2–73.5	14 (23)	3 (7)	0.056	0.001 14.3; 1.8–113	0.332
Peripheral enthesitis	7 (14)	15 (15)	0.964	10 (16)	6 (12)	0.583	0.761	0.767
Dactylitis	0	7 (7)	0.097	6 (10)	1 (2)	0.236	0.032 1.1; 1.02–1.2	0.462
At least one rheumatological manifestation	10 (20)	51 (50)	<0.001 3.6; 1.7–8.5	36 (59)	15 (36)	0.020 2.6; 1.1–5.8	<0.001 5.6; 2.3–13.3	0.157
Familial uveitis	2 (4.1)	7 (6.8)	0.719	6 (9.8)	1 (2.4)	0.236	0.295	1.0
Familial ankylosing spondylitis	0	11 (11)	0.017 1.12 (1.05–1.12)	11 (18)	0	0.003 1.2; 1.08–1.4)	0.001 1.2; 1.1–1.4	-
Familial SpA	8 (16.3)	41 (40)	0.004 3.4; 1.4–7.9	26 (43)	15 (36)	0.481	0.004 3.9:1.5–9.5	0.034 2.8; 1.1–7.6
Familial skin psoriasis	6 (12)	26 (26)	0.066	14 (23)	12 (29)	0.519	0.214	0.066
Familial inflammatory bowel diseases	1 (2)	8 (7.8)	0.162	4 (6.6)	4 (9.5)	0.713	0.379	0.177
Psoriasis	1 (2)	10 (9.7)	0.106	6 (10)	4 (9.5)	1.0	0.129	0.177
Inflammatory bowel diseases	2 (4)	5 (5)	0.367	2 (2)	3 (5)	0.933	0.332	0.505
Clinical Evaluation								
	GAU	NGAU	*p*	NGAU HLA-B27+ (61)	NGAU HLA-B27− (42)	*p*	GAU vs. NGAU B27+	GAU vs. NGAUB27−
Mean LEI score	0.45 + 1.0	0.76 + 1.2	0.103	0.57 (0.97)	0.89 + 1.27	0.157	0.005	0.565
LEI > 0	12 (24.5)	37 (36)	0.159	23 (38)	14 (33)	0.650	0.139	0.364
Mean MASES score	0.35 + 1.3	1.46 + 2.6	<0.001	1.7 + 2.6	1.12 + 2.6	0.275	<0.001	0.083
MASES > 0	5 (10.2)	33 (32)	0.005 (4.1; 1.5–11.4)	23 (38)	10 (24)	0.138	<0.001 5.3; 1.9–15.4	0.096
Mean painful joints	0.31 + 1.1	0.99 + 2.5	0.019	0.6 + 1.4	1.3 + 2.9	0.110	0.019	0.322
Painful Joint > 0	4 (8.2)	26 (25.72)	0.016 (3.8; 1.2–11.6)	16 (26)	10 (24)	0.781	0.024 4.0; 1.2–12.9	0.046 3.5; 1.0–12.2
POLYarthritis (>5 involved joints)	1 (2)	7 (7)	0.436	6 (10)	1 (2)	0.238	0.129	1.0
Mean swollen joint	0	0.04 + 0.4	0.492	0.1 + 0.6	0	0.323	----	0.323
dactylitis	0	0	--	0	0	---	---	---
Mean painful enthesis/patient	0.9 (2.2)	2.4 (3.7)	0.001	2.8 (3.9)	1.6 (3.2)	0.090	0.001	0.184
Enthesis with pain > 0	14 (29)	43 (42)	0.117	28 (46)	15 (36)	0.303	0.063	0.505
Enthesis with pain > 5	3 (6)	22 (21)	0.019 4.2; 1.2–14.7	14 (23)	8 (19)	0.635	0.017 4.6; 1.2–16.9	0.104
Swollen enthesis	0	0	--	0	0	--	---	--
Patients with > 0 peripheral manifestations	14 (29)	48 (47)	0.035 2.2; 1.05–4.5	33 (54)	15 (36)	0.527	0.028 2.4; 1.1–5.3	0.155
Patients with ASAS axSpA	0	40 (40)	<0.001 1.6; 1.4–1.9	36 (59)	4 (9.5)	<0.001 (13.7; 4.3–43.2)	<0.001 2.4; 1.8–3.3	0.042 1.1; 1.0–1.3
Patients with ASAS pSpA	0	29 (28)	<0.001 1.4; 1.2–1.6	23 (38)	6 (14)	0.009 (3.6; 1.3–9.9)	<0.001 1.6; 1.3–1.9	0.008 1.2; 1.0–1.3
ASAS SpA-positive patients	0	45 (44)	<0.001 1.8; 1.5–2.1	36 (59)	9 (21)	<0.001 5.3 (2.1–12.9)	<0.001 2.4; 1.8–3.3	<0.001 1.3; 1.1–1.5

Data are expressed as the mean (SD) or number (%). GAU: granulomatous acute uveitis; NGAU: non-granulomatous acute uveitis; SpA: spondyloarthropathy; ASAS: Assessment of Spondyloarthritis International Society.

**Table 3:** Ultrasound examination.

	Total (152 Patients)	GAU (49 Patients)	NGAU (103 Patients)	*p*	NGAU HLA-B27+ (*n* = 61)	NGAU HLA-B27− (*n* = 42)	*p*	GAU vs. NGAU B27+	GAU vs. NGAU B27−
Patients with enthesophyte, *n* (%)	139 (91.4%)	44 (89%)	95 (92.2%)	0.111	41 (97.5%)	54 (88.5%)	0.137	0.038	0.832
Mean enthesophyte/ patient	3.5 (2.2)	3.1 + 1.9	3.6 + 2.4	0.209	4.3 + 2.4	3.2 + 2.2	0.024	0.016	0.894
Patients with entheseal erosion, *n* (%)	12 (7.9)	0 (0)	12 (11.9%)	0.009	5 (11.9%)	7 (11.9%)	1.0	0.018	0.016
Mean eroded enthesis/patient	0.10 (0.38)	0	0.16 (0.46)	<0.001	0.14 (0.42)	0.16 (0.49)	0.820	0.032	0.011
Patients with entheseal hypoechogenicity, *n* (%)	13 (8.6%)	4 (8.2%)	9 (8.9%)	1.0	5 (11.9%)	4 (6.8%)	0.481	0.544	1.0
Mean enthesis with hypo/patient	0.14 (0.53)	0.08 + 0.3	0.17 + 0.6	0.182	0.26 + 0.83	0.10 + 0.4	0.239	0.184	0.803
Patients with thickened enthesis, *n* (%)	140 (92.7%)	19 (95%)	96 (98%)	0.440	37 (97.4%)	59 (97.3%)	1	1	0.440
Patients with Doppler signal at enthesis, n (%)	37 (24.2%)	6 (12.2%)	31 (28.7%)	0.025	13 (31%)	18 (27.1%)	0.875	0.023	0.029
Mean entheses with PD/patient	0.8 (1.7)	0.2 + 0.58	0.9 + 1.8	<0.001	0.5 + 1.1	0.7 + 1.5	0.377	0.006	0.003
Patients with acute enthesitis, *n* (%)	17 (11.2%)	2 (4.1)	15 (14.9%)	0.058	7 (16.7%)	8 (13.6%)	0.778	0.159	0.180
Mean number of acute entheses/patient	0.14 (0.52)	0.04 + 0.2	0.19 + 0.6	0.011	0.19 + 0.4	0.2 + 0.7	0.960	0.053	0.103
Patients with active enthesitis, *n* (%)	28 (18.4)	4 (8.2)	24 (23.3)	0.026	11 (26.2)	13 (21.3)	0.519	0.032	0.127
Mean number of active enthesis/patient	0.26 (0.69)	0.10 (0.34)	0.34 (0.79)	0.006	0.40 (0.83)	0.29 (0.76)	0.489	0.033	0.084
Patients with enthesis with structural damage, *n* (%)	137 (91.3%)	44 (89.9%)	95 (92.2%)	0.616	41 (97.6%)	54 (88.5%)	0.137	0.038	1.0
Mean enthesis with structural damage/patient	3.6 (2.3)	3.1 + 1.9	3.7 + 2.4	0.083	4.4 + 2.5	3.4 + 2.4	0.018	0.008	0.605
Mean GUESS score	8.9 (19.2)	7.1 (3.8)	9.7 (5.5)	<0.001	10.5 (5.2)	9.2 (5.7)	0.269	<0.001	0.023
Mean MASEI score	19.2 (13.6)	11.7 (7.4)	22.7 (14.4)	<0.001	25.9 (14.5)	20.4 (14.0)	0.057	<0.001	<0.001

Data are expressed as the mean (SD) or number (%). GAU: granulomatous acute uveitis; NGAU: non-granulomatous acute uveitis; GUESS: Glasgow ultrasound enthesitis scoring system; MASEI: Madrid sonography enthesitis index.

**Table 4:** Concordance between two subsequent ultrasound examinations.

	ICC	95% CI	Sign
Eroded enthesis	0.917	0.84–0.96	<0.001
Patient with eroded enthesis	0.946	0.9–0.97	<0.001
Enthesis with edema	0.673	0.37–0.83	<0.001
Patient with edematous enthesis	0.499	0.15–0.65	0.040
Power Doppler-positive enthesis	0.934	0.87–0.97	<0.001
Patients with PD-positive enthesis	0.848	0.73–0.89	<0.001
Acute enthesis	0.706	0.5–0.84	<0.001
Patients with acute enthesis	0.806	0.63–0.9	<0.001
Active enthesis	0.919	0.84–0.96	<0.001
Patients with active enthesis	0.829	0.67.0.91	<0.001

ICC = intraclass correlation coefficient; 95% CI = 95% confidence interval.

## Data Availability

The raw data supporting the conclusions of this article will be made available by the authors on request.
